# Detection of differentially expressed genes between Erhualian and Large White placentas on day 75 and 90 of gestation

**DOI:** 10.1186/1471-2164-10-337

**Published:** 2009-07-26

**Authors:** Quan-Yong Zhou, Ming-Di Fang, Ting-Hua Huang, Chang-Chun Li, Mei Yu, Shu-Hong Zhao

**Affiliations:** 1 Key Laboratory of Agricultural Animal Genetics, Breeding, and Reproduction of Ministry of Education & Key Laboratory of Swine Genetics and Breeding of Ministry of Agriculture, Huazhong Agricultural University, Wuhan, 430070, PR China; 2 Institute of Animal Husbandry and Veterinary, Jiangxi Academy of Agricultural Science, Nanchang, 330200, PR China

## Abstract

**Background:**

Placental efficiency is strongly associated with litter size, fetal weight and prenatal mortality. Together with its rapid growth during late gestation, the Large White pig breed shows a significant increase in placental size and weight, but this does not occur in the highly prolific Chinese pig breeds. To understand the molecular basis of placental development during late gestation in Chinese indigenous and Western breeds with different placental efficiency, female placental samples were collected from six pregnant Erhualian gilts at gestation day 75 (E75) and day 90 (E90) and from six pregnant Large White gilts at gestation day 75 (L75) and day 90 (L90). Two female placentas from one sow were used to extract RNA and then pooled in equal volumes. Twelve pooled samples were hybridized to the porcine Affymetrix GeneChip.

**Results:**

A total of 226 and 577 transcripts were detected that were differentially expressed between E75 and L75 and between E90 and L90 (p < 0.01, q < 0.2), respectively. Gene Ontology (GO) analysis revealed that these genes belong to the class of genes that participate in angiogenesis and development. Real-time RT-PCR confirmed the differential expression of eight selected genes. Significant differential expression of five genes in the *VEGF *pathway was also detected between the breeds. A search of chromosomal location revealed that 44 differentially expressed genes located to QTL regions related to reproduction. Differential expression of six candidate imprinted genes was also confirmed. Three of the six genes (*PLAGL1*, *DIRAS3*, and *SLC38A4*) showed monoallelic expression in the porcine placenta.

**Conclusion:**

Our study detected many genes that showed differential expression between placentas of two divergent breed of pigs, and confirmed the imprinting of three genes. These findings help to elucidate the genetic control of placental efficiency and improve the understanding of placental development.

## Background

The ratio of birth weight to placental weight can be used as a measure of placental efficiency [1]. It is determined by many factors, including the thickness and surface area of the placenta, its vascular density, and the number and activity of transporters [2,3]. In eutherian mammals, intrauterine growth shows a balance between fetal growth and the placental supply of nutrients and oxygen [4]. Fetal body weight is correlated positively with placental weight. In fact, birth size affects the long-term health of an individual, and is critical in determining life expectancy. In humans, smaller neonates are less likely to survive at birth and have a greater susceptibility to disease [5]. Thus, exploration of the genetic factors that regulate placental efficiency is an important research area.

The porcine placenta is chorioallantoic; it originates from the trophoblast and inner cell mass with no trophoblastic invasion of uterine vessels [6]. It is responsible for the exchange of respiratory gases, nutrients, and waste products between the maternal and the fetal systems. In fact, prenatal fetal organs do not participate in any nutrient metabolic pathways; all their metabolic demands are supplied by transplacental exchange from mother to fetus [7]. The placenta is a provisional organ, which only emerges during gestation. It secretes a variety of steroid and protein hormones that act in a paracrine manner on the endometrium and fetus to elicit changes in gene expression that support the growth and development of the conceptus.

High placental efficiency is thought to allow smaller placentas to maintain relatively larger fetuses, thereby contributing to higher uterine capacity and litter size [8,9]. In fact, placental insufficiency is the primary mechanism through which intrauterine crowding of fetuses decreases fetal survival [10]. In the pig, the period from gestation day 75 to gestation day 90 is an important stage for placental and fetal development, during which the fetuses grow rapidly and need adequate nutrition. Vonnahme et al. [11] found that if one of two adjacent fetuses dies in the uterus, in the Meishan pig the other conceptus fails to increase its placental weight or surface area. In contrast, the adjacent conceptus of a Large White pig accelerates its placental growth. This indicates that Meishan and Large White conceptuses use different strategies in the competition for nutrients during gestation. The Meishan increases the vascular density of the placenta in order to obtain adequate nutrition, while the Large White conceptus accelerates its placental growth instead of enhancing placental efficiency [12]. Previous studies that involved translocation of embryos from Meishan to Large White sows indicated that the number of conceptuses transferred at gestation day 30 is generally reduced during late gestation as a result of the limited ability of the uterus to maintain a large number of fetuses to term [13]. It will be of great interest to understand the molecular mechanism underlying the differences in placental gene expression between Chinese indigenous and Western breeds of pig.

In this study, we investigated differences in the expression profiles of placental genes between Erhualian and Large White pigs in late gestation by use of the Affymetrix GeneChip. The Erhualian is a type of Taihu breed, and has larger litters than the Meishan. Bioinformatics analysis revealed that the differentially expressed genes are involved in important biological processes such as angiogenesis and embryonic development in utero. Real-time RT-PCR was used to confirm the differential expression of various genes. Significant differential expression of five genes in the *VEGF *pathway was also detected between Large White and Erhualian pigs using real-time RT-PCR. The differentially expressed genes were also mapped to QTL regions related to reproduction traits such as number born alive, fully formed piglets, number of stillborn piglets, etc. Differential expression of candidate imprinted genes was also detected, and the imprinting status of three genes was determined. Our results offer new insight towards an understanding of the molecular basis of placenta efficiency.

## Results

### Transcriptomes in the placenta

**Figure 1:**
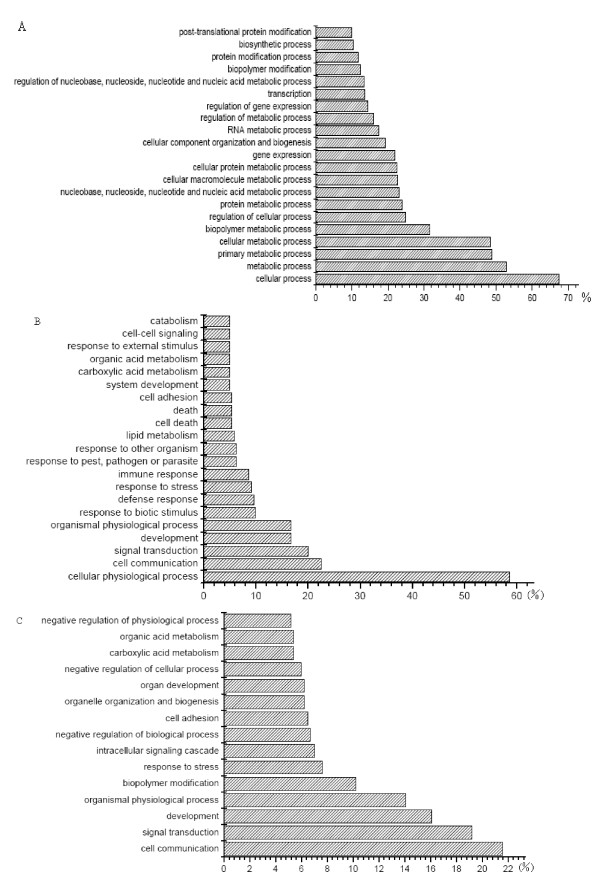
**Gene Ontology (GO) molecular function classification of all genes expressed in placenta and differentially expressed genes**. (A) All expressed genes in placenta. (B) Differentially expressed genes in E75 vs L75. (C) Differentially expressed genes in E90 vs L90. The x-axis shows the gene percentage of each GO category with regard to the placenta transcripts or the declared differentially expressed genes and the y-axis represents each GO category.

There are 24,123 probesets, which represent 20,201 transcripts and 198 controls, on the Affymetrix porcine GeneChip. The transcriptome analysis indicated that 7,842 probesets were expressed in the placenta (GEO accession numbers: GSM299411-GSM299422). Through BLAST sequence similarity analyses, 6087 transcripts were matched to human Refseq entries. GO annotation was used to classify these genes into groups representing different biological processes. The results revealed that these genes were involved in metabolic processes, transport and developmental process, etc. (Figure 1A). It is interesting that many genes had high and stable expression in E75, E90, L75 and L90. The strong expression of these genes in the placenta suggests that they may have important roles in placental development or nutrient transportation. We further checked the relative expression of some homologous genes in data obtained from human . Some genes that are specifically expressed in the placenta (*EBI3*, *HSD3B1*, *HSD17B1*), or are specifically expressed in a few tissues including the placenta, in other species [14], such as *FOXF2*, *COL1A1*, *COL3A1*, *PERP*, *SPP1 *and *PAPPA*, were detected to have high expression in our study.

### Identification of differentially expressed genes

We identified 226 (p < 0.01 and q < 0.2) transcripts that were differentially expressed between E75 and L75 (see additional file 1). Among these genes, 160 transcripts were matched to human Refseq entries through BLAST sequence similarity analyses, while the other 29% of the transcripts were non-annotated. Of the differentially expressed genes, 94 transcripts were expressed at higher levels in E75, while 132 transcripts were expressed more abundantly in L75.

Between E90 and L90, 577 (p < 0.01 and q < 0.2) differentially expressed transcripts were found (see additional file 2). Among these genes, 435 transcripts were matched to human Refseq entries through BLAST sequence similarity analyses, while the other 25% of transcripts were non-annotated. In total, 277 transcripts were expressed at higher levels in E90, and 300 transcripts were expressed more abundantly in L90. A total of 102 transcripts were detected to have differential expression both in E75 vs L75 and in E90 vs L90. But only 96 transcripts had the same expression trends in pigs of the two gestational ages.

### Real-time RT-PCR verification of differentially expressed genes

**Figure 2:**
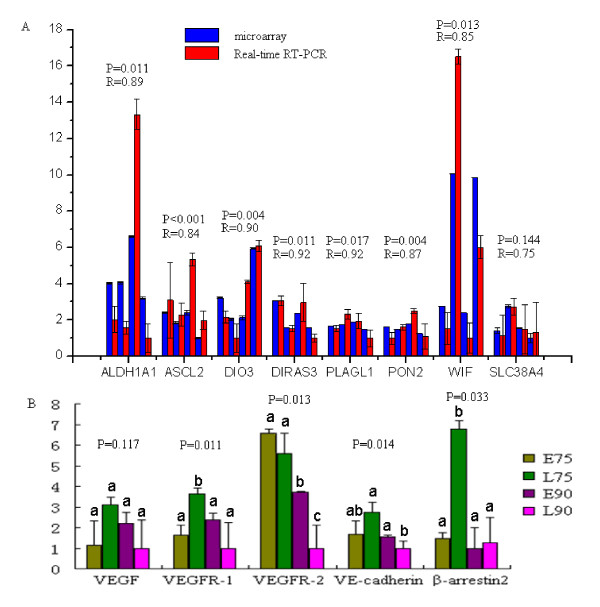
**Real time RT-PCR results of 13 genes**. (A) Validation of the Microarray data by real-time RT-PCR analysis of eight representative genes. Expression levels of eight genes were detected in E75, L75, E90 and L90 by real-time RT-PCR (red) and microarray (blue). R represents the Pearson correlation coefficient. (B) real-time RT-PCR results of genes related to vascular development and vascular permeability. The x-axis represents the genes and the y-axis shows the fold change in expression. Different superscript alphanumeric characters indicate a statistically significant difference at p < 0.05.

Thirteen genes (*ALDH1A1*, *DIO3*, *DIRAS3*, *PLAGL1*, *PON2*, *ASCL2*, *WIF1*, *SLC38A4*, *VEGF*, *VEGFR-1*, *VEGFR-2*, *VE-cadherin *and *β-arrestin2*) were selected to confirm the expression by the use of real-time RT-PCR. Among these genes, eight genes were used to validate the microarray data. The results indicated that expression patterns of these genes were consistent with the microarray (Pearson correlation coefficient >0.75, Figure 2A). The other five genes play important roles in placental angiogenesis and vascular permeability, so we also detected their expression by real-time RT-PCR. Four genes showed more than two-fold differences between breeds in the microarray analysis, but were not statistically significant. Gene *β-arrestin2 *is another important gene in the *VEGF *pathway but is not on the microarray. Expression of *VEGFR-1 *was significantly lower in E75 than L75, but significantly higher in E90 than L90. *VEGFR-2 *showed significantly higher expression in E90 than in L90. The expression levels of the *VE-cadherin *and *β-arrestin2 *genes were significantly lower in L90 than L75.

### Cluster analysis

**Figure 3:**
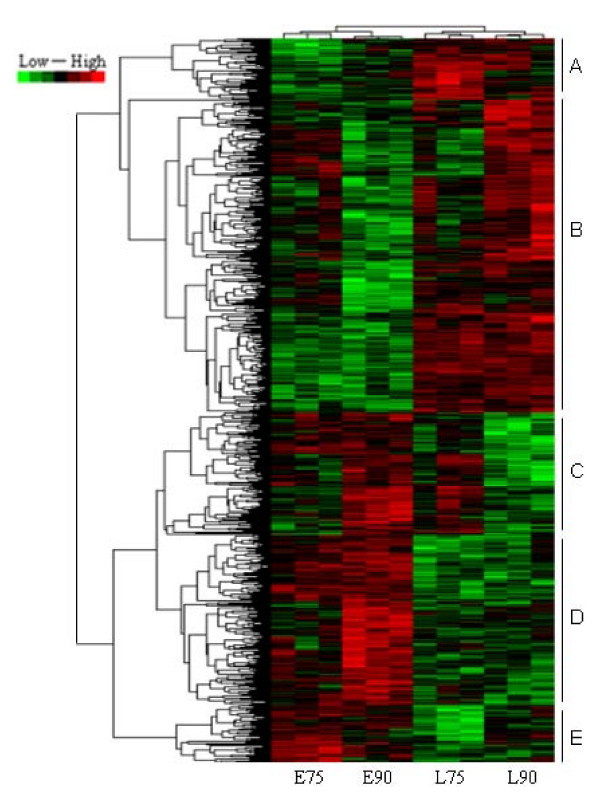
**Hierarchical cluster of differentially expressed genes**. We have performed a data adjustment (median center and normalization) in the cluster analysis. The color codes of red, white, black and dark green represented expression levels of high, average, low and absent respectively. A detailed view of the genes expression levels in clustering patterns is shown in the plot areas from A to E.

To gain insight into similarities at the transcriptome scale among placentas from conceptuses of two breeds and two ages, data from all the differentially expressed genes in the placentas were used in a systematic cluster analysis. The results showed that L75 and L90 were initially clustered together because their expression profiles were similar; E75 and E90 were clustered in another class (Figure 3).

### GO analysis

GO classification on the basis of biological process was used to categorize 226 genes that were differentially expressed between E75 and L75. The results indicated that the proteins encoded by these genes are associated with metabolic process (56.5%), biological regulation (32.5%), developmental process (21.4%) and electron transport (5.2%) (Figure 1B). The GO biological process classification of 577 genes that were differentially expressed between E90 and L90 indicated that more genes encoded proteins associated with cellular process (65.5%), metabolic process (51.0%), developmental process (22.6%) and transport (16.2%) (Figure 1C).

### Expression analysis of candidate imprinted genes

Imprinted genes, which are expressed in a monoallelic fashion depending on their parental origin, play important roles in mammalian fetal development, growth and behavior. They also influence the growth of the placenta. A homolog search using the available sequences of imprinted genes in the human and mouse genomes  identified 19 candidate imprinted genes on the porcine Affymetrix Genechip (see additional files 3 and 4). Eight candidate imprinted genes showed differential expression between the two breeds in the microarray analysis. One genes (*DIRAS3*) had significantly higher expression in E75 than in L75, and seven genes (*PON2*, *PLAGL1*, *DCN*, *DIO3*, *NAP1L5*, *ASCL2*, *SDHD*) showed significant differential expression between E90 and L90.

**Figure 4:**
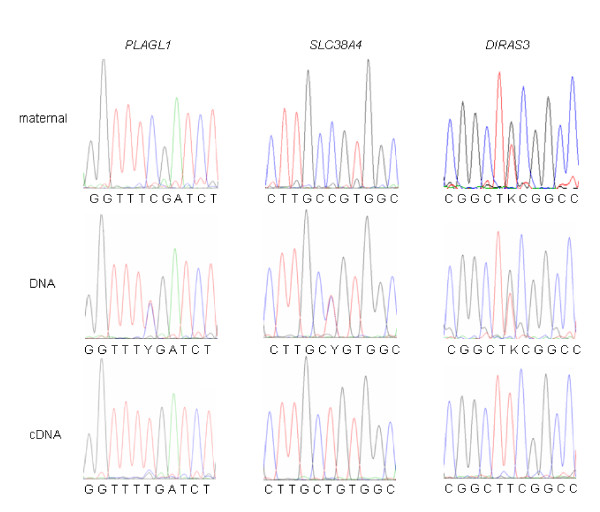
**Imprinting status of *PLAGL1*, *SLC38A4 *and *DIRAS3 *genes in porcine placentas**. The results indicated that *PLAGL1 *and *SLC38A4 *genes are paternal expression in porcine placenta. *DIRAS3 *gene is monoallelic expression in porcine placenta.

To further investigate the allelic expression of candidate imprinted genes in the porcine placenta, cDNA sequences of from both breeds were compared. In the *PLAGL1 *gene, one single nucleotide polymorphism (SNP) (T/C) was detected at position 1428 (FJ746562). In the *SLC38A4 *gene, an SNP (C/T) was detected at position 21 (FJ746563), and in the DIRAS3 gene, one SNP (T/G) was revealed at position 428 (NM_001044598). Restriction enzyme digestion (*Taq*I, *Btg *I, and *Apek *I) were used to digest the genomic DNA and cDNA of placentas. At least three heterozygotes were used to confirm the expression of imprinted genes, and the results showed that *PLAGL1 *and SLC38A4 were paternal expressed. Unfortunately, we could not detect the parental source of *DIRAS3 *gene, because that maternal genetype also was heterozygotes (Figure 4). No cSNPs were detected in other candidate imprinted genes, thus the imprinting status was not determined for the remaining genes.

### In silico mapping of differentially expressed genes to reproduction QTL regions

Through *in silico *analysis, 44 differentially expressed transcripts were found in porcine reproduction QTL chromosomal regions (see additional file 5). The traits associated with these QTL regions included ovulation rate (33 transcripts), age at puberty (8 transcripts), and uterine capacity (3 transcripts)  (see additional file 5).

## Discussion

During late gestation, Erhualian and Large White sows show different uterine responses to the rapid growth of the fetuses. For example, the placenta of the Erhualian pig stops growing, whereas Large White pigs continue to increase the surface area of the placenta [12,15,16]. This indicates indirectly that there may be many genes involved in the differences in placental development between Erhualian and Large White pigs (Table 1).

**Table 1:** Candidate genes may have important function in placenta.

Gene	Fold change		Function	Ref	p value	q value
					
	E75/L75	E90/L90				
*HOXA13*		1.45	angiogenesis	30	P < 0.001	q = 0.05
*TSP*-1	0.50		angiogenesis	31	P < 0.001	q = 0.09
*HAND2*	4.61		angiogenesis	32	P = 0.009	q = 0.19
*GR*		3.10	increase concentrations of glucose	36,37	P < 0.001	q = 0.03
*PTGS1*	3.06	3.94	glucocorticoid responsive elements	38	P = 0.001	q = 0.06
*MED14*		1.45	GR coactivators	39	P = 0.005	q = 0.17
*CHD8*	0.23	0.34	embryonic development	42	P < 0.001	q = 0.02
*FUT8*		2.70	embryonic development	43	P = 0.015	q = 0.23
*PREP1*	1.62		embryonic development	44	P < 0.001	q = 0.05
*SLC16A10*	3.09	3.75	transporter member	45	P < 0.001	q = 0.04
*SLC2A12*		3.85	transporter member	46	P = 0.046	q = 0.33
*SLC25A24*		1.67	transporter member	47	P < 0.001	q = 0.07
*SLC1A1*		1.90	transporter member	48	P = 0.009	q = 0.19
*SLC20A1*	0.17	2.41	transporter member	-	P < 0.001	q = 0.05
*DIRAS3*	2.60	2.96	inhibits growth	53	P = 0.030	q = 0.09
*PLAGL1*	0.65	1.94	growth retardation	54	P = 0.013	q = 0.22
*DCN*		0.13	placental development	58	P = 0.019	q = 0.25

The "Ref" is the number of the paper in the **Reference**.

### Genes involved in vascular development and permeability pathway

Placental vascular development and permeability are important in transportation of nutrients, respiratory gases, and waste products between the maternal and fetal circulations. In many cases, to keep pace with fetal growth, uterine and umbilical blood flow increases markedly during late gestation [17-19]. Insufficient blood flow leads to nutritional deficiency, and compromises fetal growth, which affects not only the neonate but also the health and productivity of the resulting adult throughout life [20]. Thus it is important to investigate the expression of genes in the *VEGF *pathway in order to understand placental efficiency. The results of this study showed that the expression of these genes was significantly decreased from L75 to L90, while no significant changes were found between E75 and E90 (except in *VEGFR-2*). This indicates that vascular development and permeability may be stable in E75 and E90, but that the Large White showed notable variation accompanying further development of the placenta during this period.

Vascular endothelial growth factor (*VEGF*) is produced and secreted in the placenta; it serves as an angiogenic and permeability enhancing factor in several species, including the pig [21]. It has two specific receptors (*VEGFR-1 *and *VEGFR-2*). The interaction of *VEGF *and *VEGFR-2 *could improve the mitogenic ability of *VEGF*, and lead to survival, migration, and differentiation of endothelial cells, and mediation of vascular permeability [22]. Compared with *VEGFR-1*, *VEGFR-2 *plays a major role in the biological activity of *VEGF *[23]. A number of signal transduction pathways downstream of *VEGFR-2 *play important roles, especially in vasculogenesis. In the results of our real-time RT-PCR, the expression of *VEGFR-2 *had no significant difference between E75 and L75, but was significantly higher in E90 than in L90. This indicates that the Erhualian placenta maybe have higher blood vessel density comparing with the Large White placenta. Thus Erhualian pigs can provide sufficient nutrients despite a markedly smaller placenta. Of interest, the expression of *VEGFR-1 *in the E75 placenta was lower than that in the L75, but at gestation day 90 in the Large White placenta, this gene was down-regulated and showed no significant difference from that of the Erhualian. *VEGFR-1 *may decrease the binding of *VEGF *to *VEGFR-2 *by sequestration of *VEGF*, thereby preventing its binding to *VEGFR-2 *[24]. Of course, *VEGFR-1 *and -2 may also form heterodimers whose signal efficiency is equal to or even greater than that of the *VEGFR-2 *homodimer [25]. Even so, recent data indicate that *VEGFR-1 *plays a positive regulatory role in vascular permeability [26]. *VE-cadherin1*, a cell-cell adhesion molecule specific to the endothelium, also interacts with *VEGFR-2*. This complex may promote cell internalization, lead to decomposition of intercellular junctions, and promote endothelial permeability [27]. *β-arrestin 2 *also aids endocytosis of *VE-cadherin*. A knockdown experiment indicated that *β-arrestin 2*^-/- ^inhibits the effect of *VEGF *on both endocytosis of *VE-cadherin *and endothelial permeability [28]. The expression of *β-arrestin 2 *and *VE-cadherin *in placentas from Erhualian pigs was lower than in Large White placentas on day 75 of gestation, but there was no significant difference on day 90.

Various genes related to vascular development were also detected in the microarray analysis. *HOXA13*, which is essential for placental vascular patterning and labyrinth endothelial specification, showed no differential expression between breeds at gestation day 75, but had higher expression in E90 than in L90 [29]. *TSP-1*, which is a negative regulator of angiogenesis, showed higher expression in L75 than in E75 [30]. *HAND2 *is required for vascular development and regulation of angiogenesis [31]. It was expressed at a higher level in the Erhualian than the Large White. All these results indicate that the vascular development and permeability of the placenta is different in the Erhualian and Large White. During placental development in the Large White, this difference increases. This may explain the different response of the placenta of Erhualian and Large White pigs to rapid fetal development in late gestation.

### Hormone related genes

The placenta is an endocrine organ which synthesizes many kinds of hormones that regulate its development and alter maternal physiology to support gestation. It can influence and regulate the expression of the fetal hormone genes [32]. Regulation of endocrine signals in the placenta, especially in late gestation, may over-ride maternal signals, owing to the commencement of accelerated growth of the fetus. This parasecretion may lead to the death of both mother and offspring [33]. In our results, some specific hormone related genes were shown to have differential expression between the two breeds of pig. Glucocorticoids are steroid hormones that play important roles in metabolic, immunological and development, in particular in stimulation of several processes that collectively serve to increase and maintain concentrations of glucose in blood [34]. They exert their actions by binding to the complexes which is composed with hormone-receptor and glucocorticoid responsive elements complexes [35]. In our experiment, we found that the level of expression of *GR *(glucocorticoid receptor) was higher expression in E90 than L90. The *PTGS1 *gene, which is a glucocorticoid responsive element, also could regulate angiogenesis in endothelial cells [36,37]. Its expression level was both higher in E75 and E90 than L75 and L90. *MED14 *(Mediator complex subunit 14), which is the GR coactivators, also showed higher expression in E90 than in L90 [38]. All these results showed that the Erhualian placenta may be more efficiently to able obtain glucose from the uterus than that of the Large White. Moreover, the level of expression of the. estrogen receptor 1, mineralocorticoid receptor, growth hormone regulated TBC protein 1, thyroid hormone receptor associated protein 2 and thyroid hormone receptor associated protein 3 all were significantly higher in the Erhualian than in the Large White at the same stage of gestation. All these differentially expressed genes are major regulators of the function of hormone gene. Therefore, we presume that the Erhualian placenta can provide an environment that supports optimal absorption of nutrients. The placenta of this breed may be able to extract nutrition from the uterus more efficiently than that of the Large White.

### Candidate genes for placental development and nutrient transportation

A reduction in placental size may mediate negative regulation of maternal nutrition on the birth weight and the number of secondary muscle fibers in the offspring [39,40]. During late gestation, the Large White placenta increases in size and surface area. In contrast, the Erhualian placenta remains unchanged in size and weight [12]. The genes that are involved with placental development are poorly understood. In our study, the expression of the gene *CHD8 *was higher in the Large White than in the Erhualian on gestation day 75 and 90. In the mouse, Nishiyama et al. [41] found that *Chd8 *could inhibit p53-dependent apoptosis through binding to p53. Chd8(-/-) mice died early during embryogenesis. It has strong expression in the placenta and other reproductive tissues. Therefore we presume that this gene may play roles in placental development. In fact, there are some analogous genes among those investigated in our study. For example, *FUT8 *is associated with in-utero development of the embryo [42]. It showed very high expression in the placenta and may potentially be the genes that influence placental development. The *Prep1 *gene regulates multiple aspects of embryonic development in the mouse through a Pbx-Meis network [43]. It was also found to have higher expression in Erhualian placentas than in those of Large White pigs. The GO molecular function classification showed that the percentage of differentially expressed genes that encoded proteins associated with developmental process was 21.4% on gestation day 75 and 22.6% on gestation day 90. All these results provide indications of potential genes that warrant further study in investigating the disparity in placental development between indigenous Chinese and exotic pig breeds.

One of the primary regulators of maternofetal transfer of nutrition is the density of transporter proteins in the placenta. In our results, we also detected some genes in the solute carrier family. *SLC16A10*, which is a member of a family of plasma membrane amino acid transporters, mediates the Na(+)-independent transport of aromatic amino acids across the plasma membrane [44]. It showed up-regulation in the Erhualian placenta on both gestation day 75 and gestation day 90, in comparison to that of the Large White. *Slc2a12 *has been designated as a new member of the glucose transporter family in the mouse [45]. *SLC25A24 *plays important roles in the net uptake and efflux of adenine nucleotides [46]. *SLC1A1 *also participates in glutamate transport [47]. These genes showed no difference in expression between E75 and L75, but showed higher expression in E90 than in L90. Differential expression was also detected in genes of other solute carrier families. The *SLC20A1 *gene was expressed at higher levels in L75 than in E75, but in placentas on gestation day 90, it had higher expression in E90. All these results suggest that Erhualian placentas can supply more transporters and transport nutrients more efficiently.

### Imprinted genes

The genome of the fetus inherits two copies of most genes, one from the mother and the other from the father. Generally, the two single strands have similar transcription activities and equivalent function. However, imprinted genes escape this rule, and preferentially express maternally or paternally derived alleles. They have evolved in mammals because of the conflicting interests of maternal and paternal genes in relation to the transfer of nutrients from the mother to her offspring [48]. They play central roles in fetal growth through controlling the fetal demand for, and the placental supply of, maternal nutrients [49]. Detecting the imprinting status of genes, especially in the placenta, is very important. The information available is very useful in comparisons of mouse, human, cattle and pig genes because of the conservation of expression and function of imprinted genes [50]. In this study, we detected the imprinting status of three potentially imprinted genes in the porcine placenta. The *SLC38A4 *gene is paternal expression in the pig. This is the same as the mouse, but not cattle [51]. The *Diras3 *gene displays paternal-specific expression both in mice and humans [52]. We found that it is also expressed in a monoallelic fashion in the porcine placenta. The *PLAGL1 *gene was found to be imprinted in the porcine placenta. In the human and mouse, these genes are paternally expressed [53]. From our evaluation of the conservation of imprinting status among species, we assume that these genes have the same function and expression in the pig as in the other species. In the mouse, *Diras3 *inhibits growth, and transgenic expression of this gene produces individuals of small stature [54]. The *Plagl1 *gene encodes a growth suppressor protein that is related to developmental disorders such as growth retardation and transient neonatal diabetes mellitus (*TNDM*) [53,55]. Moreover, it regulates an imprinted gene network that is critically involved in the control of embryonic growth [56]. In our study, *DIRAS3 *had higher expression in the placentas of E75 than L75. The expression of *PLAGL1 *increased from E75 to E90, but decreased from L75 to L90. The *DCN *gene, a strong candidate for function in placental development [57], was found to have higher expression in the Large White placenta. These expression patterns may be related to the phenotype, in that the Erhualian has a smaller fetus and placenta than the Large White pig.

## Conclusion

We have reported differential gene expression in placentas at gestation day 75 and 90 from pig breeds with different placental efficiency. Cluster analysis indicated that E75 has more similarity on the transcriptome scale with E90 than with L75 and L90. This finding could indicate that from gestation day 75 to gestation day 90, Erhualian and Large White pigs show a large variance in placental development. We also identified some differentially expressed genes that are related to angiogenesis and vascular permeability, transport, fetal development and hormonal regulation. The up-regulation of genes associated with the promotion of proliferation of blood vessels and transporter proteins in Erhualian pigs may be responsible for providing sufficient nutrients to support fetal development. The results of this study provide an opportunity to elucidate the genetic control of placental efficiency and improve our understanding of placental development.

## Methods

### Animal and tissue preparation

All animal procedures were performed according to protocols approved by the Biological Studies Animal Care and Use Committee of Hubei Province, P. R. Six Erhualian and six Large White sows were mated with 6 boars of each corresponding breed. These boars and sows have no common grandparents, they can be treated as unrelated animals. Three sows of each breed were killed and necropsied on days 75 and 90 of gestation, respectively. The uterus was removed immediately and the occupied fetal placentas of each fetus were collected, washed briefly with PBS, and then snap frozen in liquid nitrogen for later experiments.

### Porcine Affymetrix GeneChip hybridization and data analysis

Two female placentas from each sow were used to extract RNA and then the samples pooled in equal volumes. In total, 12 pools were prepared from the 12 sows, i.e. three biological replicates at each stage and for each breed were prepared for hybridization. The RNAs were sent to a commercial service for hybridization to the porcine Affymetrix GeneChip. In brief, 10 micrograms of total RNA was transcribed to first- and second-strand cDNAs according to the manufacturer's instructions (Affymetrix, Inc., Santa Clara, CA). After purification and testing on an Agilent Bioanalyser 2100 machine, the double-stranded cDNA served as a template for the in vitro transcription reaction for cRNA amplification. The cRNA was labeled with biotin using the GeneChip One-Cycle target labeling kit (Affymetrix; Expression Analysis Technical Manual). The labeled cRNA was quantified, fractionated, and hybridized with the GeneChip Porcine Genome Array according to the standard procedures provided by the manufacturer. Chips were washed and stained with a GeneChip Fluidics Station 450 (Affymetrix). The chips were then scanned with an Affymetrix GeneChip Scanner 3000 (Affymetrix).

Data from the cel files were converted to gene signal files by MAS 5.0 (microarray analysis system 5.0, Affymetrix) default normalization. The data were normalized between slides using the quantile normalization method proposed by Bolstad et al. [58] in R software. The natural logarithm was taken for measures of expression for each chip and used for the next step of the analysis. Differentially expressed genes were identified by analyzing these normalized data using a general linear model in SAS (SAS Institute, Cary, NC) on a gene by gene basis. The model for this experiment was Y_ijk _= μ + B_i _+ D_j _+ BD_ij _+ E_ijk_. Y_ijk _is the base-e logarithm of the normalized measurement of expression level from breed i (i = 1, 2), pregnancy day j (j = 1, 2), and individual k (k = 1, 2, 3). In the equation, μ represents an overall mean value, B is the main effect for breed, D is the main effect for day of gestation, BD is the interaction effect of breed and day, and E is the stochastic error. The q values were calculated using the method of Storey and Tibshirani [59]. An estimate of the upper bound of the positive false discovery rate was represented by the largest q value in a list of genes identified to be differentially expressed. Expression on the Affymetrix GeneChip porcine genome array probeset was determined by comparing the expression signals of perfect match (PM) and mismatch (MM) 25-mer probes. In our data set, if there is more than two P (present) in the triplicate detection data for E75, L75, E90 or L90 for a probeset, then we count this gene as expressed in the placental transcriptome.

Cluster analysis and annotation of gene ontology Hierarchical cluster analysis of differentially expressed genes in the placentas was conducted using Gene Cluster 3.0 and tree view software (Stanford University, 2002). The Database for Annotation, Visualization and Integrated Discovery (DAVID 2.0 and 2.1 beta) provides a comprehensive set of tools to summarize gene annotation data visually ([60]; ). Functional annotations were pursued for the differentially expressed genes selected. The TC accession numbers were first updated from TIGR 5.0 to TIGR 11.0 and the corresponding Human Gene IDs were pulled out so that the DAVID analysis software could be interrogated. The data were then analyzed for each of the above comparisons using DAVID 2.0 and 2.1 beta.

### Real-time RT-PCR confirmation of differentially expressed genes

Real-time RT-PCR was used to verify the differential expression of eight genes that were detected by the Affymetrix GeneChip. The primers used are listed in additional file 6. Each real-time RT-PCR reaction (in 25 μL) contained 2×SYBR Green Realtime PCR Master Mix, 0.4 μM primers, and 0.5 μL of template cDNA. The cycling conditions consisted of an initial, single cycle of 5 min at 95°C, followed by 40 cycles of 30 sec at 95°C, 30 sec at 60°C, 15 sec at 72°C, and fluorescence acquisition at 83°C for 1 sec. The cDNA was synthesized using reverse transcriptase (Promega), oligo(dT) and random primers with 5 μg RNA treated with DNase I (Ambion, Austin, Texas) from the same placental samples as those used in the microarray. The PCR amplifications were performed in triplicate for each sample. The gene expression levels were quantified relative to the expression of RPL32 using Gene Expression Macro software (Bio-Rad, Richmond, CA), by employing an optimized comparative Ct (ΔΔCt) value method. Dissociation curves were generated to ensure that a single amplicon was produced for each gene. The differences in gene expression levels between groups were compared using the Student's t-test. A p value < 0.05 was considered significant. The Pearson correlation coefficient was calculated and used to estimate the correlation of real time RT-PCR results and microarray results.

### Detection of imprinting

The differentially expressed candidate imprinted genes were further investigated for their imprinting status in the placental samples collected in this study. The unigene mRNA and expressed sequence tags (EST) were assembled to produce a contig, and then used to design primers using Primer 5.0. Sequencing of the PCR products from Erhualian and Large White pigs was used to detect SNPs in the cDNA region. Restriction fragment length polymorphisms (RFLP) were used to confirm the SNPs. The PCR products were separated in 2% agarose gels containing 0.5 g/ml ethidium bromide after digestion by restriction enzymes. All genomic DNA obtained from 132 piglets was used to detect heterozygous animals. Total RNA from the placenta of a heterozygous fetus was treated with the TURBO DNA-free kit (Ambion, Austin, USA) and was reverse transcribed to cDNA. The primer pairs shown in Table 1 were employed to amplify the genomic DNA and cDNA from the same heterozygous samples. The amplicons were digested with restriction enzymes. Sequencing was further conducted to confirm the genotype. The epigenetic status was determined by comparing the allelic expression of genomic DNA and cDNA from the same samples.

### Integration of differentially expressed genes in porcine QTL regions

The Pig Quantitative Trait Loci (QTL) database  has gathered all pig QTL data published during the past 10 years. The reproductive traits include total number born, total number born alive, fully formed piglets, number of stillborn, ovulation rate, age at puberty and uterine capacity. We downloaded all Affymetrix probesets that were located in these QTL regions. The differentially expressed probesets located in these regions were identified through comparison analysis.

## Abbreviations

APPA: pregnancy-associated plasma protein A; ALDH1A1: aldehyde dehydrogenase family 1, subfamily A1; ASCL2: achaete-scute complex homolog 2; CHD8: chromodomain helicase DNA binding protein 8; COL1A1: collagen, type I, alpha 1; COL3A1: collagen, type III, alpha 1; DCN: decorin; DIO3: deiodinase, iodothyronine type III; DIRAS3: DIRAS family, GTP-binding RAS-like 3; EBI3: Epstein-Barr virus induced gene 3; ESR1: estrogen receptor 1; EST: expressed sequence tags; FOXF2: forkhead box F2; FUT8: fucosyltransferase 8; GO: Gene Ontology; GR: glucocorticoid receptor; HAND2: heart and neural crest derivatives expressed 2; HOXA13: homeo box A13; HSD17B1: hydroxysteroid (17-beta) dehydrogenase 1; HSD3B1: hydroxy-delta-5-steroid dehydrogenase, 3 beta- and steroid delta-isomerase 1; IHH: Indian hedgehog homolog; NAP1L5: nucleosome assembly protein 1-like 5; PCR: polymerase chain reaction; PERP: PERP, TP53 apoptosis effector; PKNOX1: PBX/knotted 1 homeobox 1; PLAGL1: pleiomorphic adenoma gene-like 1; PON2: paraoxonase 2; PREP1: PBX/knotted 1 homeobox 1; PTGS1: prostaglandin-endoperoxide synthase 1; QTL: quantitative trait locus; SDHD: succinate dehydrogenase complex, subunit D, integral membrane protein; SLC16A10: solute carrier family 6, member 10; SLC1A1: solute carrier family 1, member 1; SLC20A1: solute carrier family 20, member 1; SLC25A24: solute carrier family 25, member 4; Slc2a12: solute carrier family 2, member 12; SLC38A4: solute carrier family 38, member 4; SNP: single nucleotide polymorphisms; SPARC: secreted protein, acidic, cysteine-rich; SPP1: secreted phosphoprotein 1; TSP-1: thrombospondin 1; VEGF: vascular endothelial growth factor; VEGFR-1: soluble vascular endothelial growth factor receptor-1; VEGFR-2: soluble vascular endothelial growth factor receptor-2; WIF1: WNT inhibitory factor 1.

## Authors' contributions

SHZ conceived the research. CCL and MY supervised the research; all authors carried out the research. MDF and THH analyzed the data. QYZ analyzed the data, carried out the real time RT-PCR, and wrote the first version of the manuscript. All authors have read and approved the final manuscript.

## Supplementary Material

Additional file 1 **Comparison of normalized expression levels between E75 and L75**. The data provided represent the normalized expression levels of E75 and L75. Statistical analysis indicated that 226 transcripts were differentially expressed between E75 and L75 (p < 0.01, q < 0.2). Click here for file

Additional file 2 **Comparison of normalized expression levels between E90 and L90**. The data provided represent the normalized expression levels of E90 and L90. Statistical analysis indicated that 577 transcripts were differentially expressed between E90 and L90 (p < 0.01, q < 0.2). Click here for file

Additional file 3 **Comparison of normalized expression levels of candidate imprinted genes between E75 and L75**. The data listed the normalized expression levels of imprinted genes in E75 and L75. Click here for file

Additional file 4 **Comparison of normalized expression levels of candidate imprinted genes between E90 and L90**. The data listed the normalized expression levels of imprinted genes in E90 and L90. Click here for file

Additional file 5 **Differentially expressed genes located in reproduction QTL regions**. The file listed all differentially expressed genes located in reproduction QTL regions. Click here for file

Additional file 6 **Primer pairs designed for genes selected for validation by real time RT-PCR and SNP detection**. The file listed all primer pairs designed for real time RT-PCR and SNP detection. Click here for file
